# Facial Emotion Recognition in Patients with Juvenile Myoclonic Epilepsy

**DOI:** 10.3390/jcm12124101

**Published:** 2023-06-17

**Authors:** Hannah Dunkel, Adam Strzelczyk, Susanne Schubert-Bast, Matthias Kieslich

**Affiliations:** 1 Clinic for Children and Adolescents, Department of Neuropediatrics, Goethe-University, 60590 Frankfurt am Main, Germanysusanne.schubert-bast@kgu.de (S.S.-B.); 2 Epilepsy Center Frankfurt Rhine-Main and Department of Neurology, Goethe-University, 60528 Frankfurt am Main, Germany; strzelczyk@med.uni-frankfurt.de

**Keywords:** facial expression recognition, Janz syndrome, genetic generalized epilepsy, social cognition, psychosocial outcome, cortical networks

## Abstract

Previous studies have found facial emotion recognition (FER) impairments in individuals with epilepsy. While such deficits have been extensively explored in individuals with focal temporal lobe epilepsy, studies on individuals with generalized epilepsies are rare. However, studying FER specifically in individuals with juvenile myoclonic epilepsy (JME) is particularly interesting since they frequently suffer from social and neuropsychological difficulties in addition to epilepsy-specific symptoms. Furthermore, recent brain imaging studies have shown subtle microstructural alterations in individuals with JME. FER is considered a fundamental social skill that relies on a distributed neural network, which could be disturbed by network dysfunction in individuals with JME. This cross-sectional study aimed to examine FER and social adjustment in individuals with JME. It included 27 patients with JME and 27 healthy controls. All subjects underwent an Ekman-60 Faces Task to examine FER and neuropsychological tests to assess social adjustment as well as executive functions, intelligence, depression, and personality traits. Individuals with JME performed worse in global FER and fear and surprise recognition than healthy controls. However, probably due to the small sample size, no significant difference was found between the two groups. A potential FER impairment needs to be confirmed in further studies with larger sample size. If so, patients with JME could benefit from addressing possible deficits in FER and social difficulties when treated. By developing therapeutic strategies to improve FER, patients could be specifically supported with the aim of improving social outcomes and quality of life.

## 1. Introduction

Juvenile myoclonic epilepsy (JME) is the most common genetic generalized epilepsy (GGE) syndrome with peak onset during puberty at a mean age of 14 years [1]. It is characterized by myoclonic jerks, which typically occur during awakenings and may be accompanied by generalized tonic-clonic seizures and/or absence seizures [2] Seizures are known to be triggered by sleep deprivation and photosensitivity is observed in 30–50% of individuals with JME [3,4]. A further characteristic of this epilepsy syndrome is the presence of interictal, brief and irregular generalized (poly-)spike-and-wave discharges with unstable frequency in electroencephalograms (EEGs) [2]. In addition to syndrome-specific symptoms, already Dieter Janz recognized neuropsychological peculiarities in individuals with JME. In one of his early reports he described their characters as unsteady, distrustful, emotionally unstable, and unreliable [5]. Since then, and increasingly in recent years, a large body of research has focused on the neuropsychological profile of individuals with JME, showing executive dysfunction, higher depression incidence, and impulsive behavior [6,7,8]. Besides syndrome-specific symptoms, these findings suggested that JME is a systemic brain disorder with underlying neuronal dysfunction [9]. While individuals with JME show no evidence of structural lesions in routine imaging, over recent decades, morphometric magnetic resonance imaging (MRI) studies have found discrete but robust microstructural abnormalities [8,10,11]. In a voxel-vise meta-analysis Kazis et al. investigated volumetric changes in the gray matter of individuals with JME compared to healthy controls. They found evidence of an increased gray matter volume in several structures (supplementary motor areas bilaterally, left median cingulate/paracingulate gyri, right superior frontal gyrus, and left precentral gyrus) as well as a volume decrease of gray matter in the left thalamus and the left insula [10]. Knake et al. used diffusion tension imaging (DTI) to assess white matter microstructure in individuals with JME compared to healthy controls. They demonstrated alterations in the white matter microstructure of individuals with JME in areas of frontal white matter bilaterally, in anterior parts of the Corpus callosum, in the cingulate gyrus, and the right thalamus. In addition they performed neuropsychological tests and showed deficits in frontal lobe cognitive performance, attention, executive functions, and short-term memory in individuals with JME [8]. Microstructural abnormalities were also found in the healthy siblings of individuals with JME, suggesting a genetically determined alteration of the brain development [12]. This theory is supported by the discovery of several JME-associated mutations in genes such as gamma-aminobutyric acid type A receptor subunit alpha1 (*GABRA1*) and EF-hand domain containing 1 (*EFHC1*) that are thought to perturb cortical development [13,14,15]. While its pathomechanism remains incompletely understood, thalamocortical network disorder with altered connectivity is now thought to be a key mechanism in JME. It could explain the generation of generalized epileptiform discharges as well as impaired frontal functions [8,10] and might also affect facial emotion recognition (FER).

FER is the ability to correctly recognize emotions from facial expressions. The six basic emotions of happiness, sadness, surprise, fear, disgust, and anger are thought to be biologically determined and universally recognizable [16]. A recent meta-analysis showed impaired FER in patients with epilepsy [17]. This impairment varies depending on the localization of the epileptic focus. Edwards et al. showed that individuals with temporal lobe epilepsy (TLE) had significant deficits in recognizing the facial emotions of fear, disgust, happiness, and anger [17]. While studies on GGE patients have reported inconsistent results, they have generally shown FER impairment specifically for fear, disgust, and anger [18,19,20]. The authors hypothesized that FER is impaired when the epilepsy focus/pathology affects structures involved in FER [17]. Emotion recognition draws on the temporal lobe’s visual processing system and a distributed network that modulates the visual system via feedback [21,22]. Therefore, the reported FER impairment in TLE patients can be explained as the pathology affects the temporal lobe. In contrast, FER impairment in GGE patients was unexpected, and the pathomechanism leading to it remains unknown. Interestingly, the authors [18,19,20] did not differentiate between the four epilepsy syndromes (JME, juvenile absence epilepsy, childhood absence epilepsy, and epilepsy with generalized tonic-clonic seizures alone), which were grouped as GGE.

To our knowledge, only one previous study has investigated FER particularly in individuals with JME. Kuchukhidze et al. assessed emotion recognition and social cognition in individuals with JME and analyzed functional MRI data. Their preliminary results of seven individuals with JME showed tendencies of deficits in executive functions and emotion recognition in neuropsychological testing and functional MRI [23]. However, more studies are needed to further investigate FER in individuals with JME and seems to be especially interesting for several reasons. Previous studies have shown microstructural alterations in JME patients that could explain impaired FER. It remains unclear whether these structural alterations also cause functional impairment [10]. In addition, individuals with JME frequently show poor social outcomes [24,25]. Since FER is considered crucial for social communication and interaction, whether impaired FER contributes to poor social outcomes is of interest. Patients could benefit from the knowledge of possible FER impairments and social difficulties since they could be specifically encouraged in this area, and treatment options could be developed.

This study’s primary aim was to determine whether individuals with JME have global or specific FER impairment. Furthermore, we assessed whether the FER ability is affected by epilepsy variables (age at seizure onset, seizure-free interval, and number of anti-seizure medication [ASM]), demographic variables (age at the time of testing), depression, or cognitive impairments. We also explored social adjustment and personality traits, as well as possible correlations with FER.

## 2. Materials and Methods

### 2.1. Subjects

This cross-sectional study included 27 patients with JME and 27 healthy controls. Patients were enrolled between September 2020 and February 2022 at the Epilepsy Center Frankfurt Rhine-Main (Frankfurt am Main, Germany). According to the International League Against Epilepsy classification guidelines [26], JME diagnoses were established through clinical diagnosis criteria and EEG recordings. The controls were healthy volunteers recruited through adverts posted at the university of Frankfurt am Main and in general practitioners offices. They were matched for sex and age (±one year). The exclusion criteria were age <16 years or >65 years, the use of >3 ASMs for epilepsy patients, structural brain damage, or the presence of comprehension deficits or learning disorders that may affect the completion of the tests or questionnaires. There was no evidence of epileptic seizures or other neurological disorders in the controls’ medical histories.

### 2.2. Ethics

All subjects provided informed written consent after receiving detailed information about the procedures and purpose of the study. This study was approved by the Ethics Committee of the Goethe University Frankfurt am Main (Protocol-No.: 19-319) and performed according to the Declaration of Helsinki.

### 2.3. Procedures

Data collection was performed according to the study protocol in a single session and took about 100 minutes. Testing was carried out for all participants under comparable conditions by one investigator. All Subjects performed a standardized test battery to evaluate FER, executive function, IQ, emotional state, social adjustment, and personality traits.

#### 2.3.1. FER

The Ekman 60 Faces Task (Ek-60FT) is a frequently used and well-validated tool for assessing emotion recognition from facial expressions. This study used a computerized version of the Ek-60FT from the Facial Expressions of Emotion—Stimuli and Tests (FEEST) [27]. It comprised a series of 60 black and white photographs of faces from the Ekman and Friesen Series “Pictures of facial Affect” [28] showing the faces of 10 different models. For each model, the series contains six images, each showing a different basic emotion (happiness, sadness, anger, surprise, disgust, and fear). The photographs are each presented for 5 seconds in a random order, and subjects are asked to assign one of the six basic emotions to every photograph. The correctly named emotions are scored, resulting in a maximum total score of 60 points. In addition, each emotion is evaluated on a 10-point subscale.

#### 2.3.2. Neuropsychological Tests

All subjects underwent a battery of standardized neuropsychological tests. To assess attention and executive functions, we used EpiTrack [29], a cognitive function screening test that is particularly sensitive to deterioration due to ASM. It comprises six subtests assessing working memory, cognitive flexibility, inhibition, processing speed, verbal fluency, and visual-spatial planning. The results are expressed as an age-corrected total score with a maximum of 42 points. A total score of <31 points indicates executive function impairment. We used the short version of the first part of the revised Culture Fair Intelligence Test (CFT-20R) [30] without repetition to asses basic intelligence. We tested social adjustment using a German computerized version of the Social Adjustment Scale—Self Report (SAS-SR) [31,32]. It assesses social adjustment in various areas of life (work, leisure and social activities, relatives, partnership, parents, family, and finances), providing total and subscale *t*-values. We used a validated German version of Becks Depression Inventory Fast Screen (BDI-FS) [33] to assess depressive symptoms. It comprises seven items: sadness, pessimism, loss of pleasure, past failure, self-dislike, self-criticalness, and suicidal ideation. Every item is rated on a four-point Likert scale (0–3 points) with a maximum total score of 21 points. A value of ≥4 indicates clinically relevant depression. All subjects completed a German revision of the NEO Five Factory Inventory (NEO-FFI) [34] to assess their personality traits. It is a shorter version of the well-known and well-validated NEO Personality Inventory-Revised (NEO-PI-R), according to Costa and McCrae [35]. The self-report questionnaire comprises 60 items and evaluates five personality traits (neuroticism, extraversion, openness to new experiences, agreeableness, and conscientiousness). The higher the determined value, the more pronounced the individual’s expression of the characteristic.

### 2.4. Statistical Analysis

The pseudonymized data analysis was performed using SPSS (version 28.0.1.1; IBM). All neuropsychological scores are expressed as mean ± standard deviation. The distributions of all variables were tested for normality using the Shapiro–Wilk test. After checking the preconditions, differences between the clinical and demographic data among the two study groups were examined by comparing means. A parametric test (two-tailed unpaired *t*-tests for independent samples) was performed for normally distributed variables (Ek-60FT total score; SAS-SR total score, and leisure/social and relatives subscale scores; IQ; Epitrack total score; and NEO-FFI). A nonparametric test (Mann–Whitney U test) was performed for non-normally distributed variables (all Ek-60FT subscale scores; SAS-SR work, partnership, parents, family, and finance subscales; and BDI-FS). Relations between the outcome variable FER (Ek-60FT total score) and the variables IQ; Epitrack total score; BDI-FS; SAS-SR total score; age at time of testing; age at seizure onset; seizure free interval; and number of ASM were analyzed by correlations. Pearson´s correlation [*r*] was used for normally distributed variables and Spearman´s rank correlation coefficient was performed for non-normally distributed variables. Given a 5% error probability, all results with *p* < 0.05 were considered statistically significant. We applied a Bonferroni correction for multiple comparisons to adjust their significance level. Statistical analyses were performed under the advice and supervision of the Institute of Biostatistics and Mathematical Modeling at The University of Frankfurt am Main.

## 3. Results

### 3.1. Subjects Characteristics

This study included 27 patients with JME (12 males [44%], mean age = 25.8 ± 5.8 years) and 27 age- and sex-matched healthy controls (12 males [44%], mean age = 26.0 ± 6.1 years). Their demographic and clinical characteristics are shown in Table 1.

### 3.2. FER

Individuals with JME performed worse than controls in FER tasks. They correctly recognized 44.7 ± 5.4 (74.6%) emotions among the 60 photographs shown in the test, compared to 47.3 ± 4.3 (78.3%) for the controls. However, this difference was not statistically significant (*t*_(52)_ = −1.93, *p* = 0.059). Compared with the norm, 9/27 (33.3%) patients scored below the cut-off of 42, indicating an impaired FER. However, 3/27 (11.1%) controls also scored below this cut-off. The results of analyzing the Ek-60FT subscales (happiness, sadness, surprise, fear, disgust, and anger) are shown in Table 2. Individuals with JME performed especially worse on FER subscales for surprise (*Z* = −2.39, *p* = 0.017) and fear (*Z* = −1.92, *p* = 0.055) when compared to healthy controls. However, these results were insignificant after the multiple testing correction. FER was not significantly correlated with age at time of testing (*r* = 0.18, *p* = 0.372), age at seizure onset (*r* = 0.01, *p* = 0.976), seizure-free interval (*r* = −0.18, *p* = 0.380), and number of ASM taken at the time of testing (*r* = −0.16, *p* = 0.417).

### 3.3. Neuropsychological Tests

The results of the neuropsychological tests evaluating executive functions, depression, social adjustment, and personality traits are shown in Table 3. The average IQ of both groups was within the normal range (patients: 98.7 ± 15.3, controls: 108.6 ± 12.5). Patients performed significantly worse in executive functions than the healthy controls (*t*_(52)_ = −3.79, *p* < 0.001). We used the BDI-FS to screen for depressive symptoms. Individuals with JME had significantly higher BDI-FS scores (4.5 ± 3.5 points) than healthy controls (1.3 ± 1.8 points; *Z* = −4.07, *p* < 0.001). FER was not significantly correlated with IQ (*r* = 0.31, *p* = 0.116), executive functions (*r* = 0.24, *p* = 0.236), or depressive symptoms (*r* = 0.14, *p* = 0.495). However, EpiTrack scores were negatively correlated with the number of ASM (*r* = −0.53, *p* = 0.005). In addition, seizure-free intervals were negatively correlated with depressive symptoms (*r* = −0.52, *p* = 0.006) and the number of ASM was positively correlated with depressive symptoms (*r* = 0.50, *p* = 0.008).

The SAS-SR test showed that individuals with JME had significantly worse social adjustment than healthy controls (*t*_(52)_ = −3.38, *p* = 0.001). The leisure and social activities scale was particularly affected (*t*_(52)_ = −4.28, *p* < 0.001). These differences remained significant after the multiple testing correction. However, FER was not significantly correlated with social adjustment in individuals with JME (*r* = −0.12, *p* = 0.555). NEO-FFI was used to examine personality traits. Individuals with JME had significantly higher neuroticism scale scores than the healthy controls (*t*_(52)_ = 4.16, *p* < 0.001). In addition, they were less extroverted (*t*_(52)_ = −4.25, *p* < 0.001) and less agreeable (*t*_(52)_ = −3.23, *p* = 0.002) than the healthy controls. While they also showed a tendency for lower openness to experience scale scores than the healthy controls (*t*_(52)_ = −2.4, *p* = 0.020), this comparison was insignificant after the multiple testing correction. No correlations were found between the extraversion, agreeableness, openness to experience, and conscientiousness scales and the epilepsy variables (age at seizure onset, seizure-free interval, and number of ASM at the time of testing). However, neuroticism scale scores correlated negatively with age at seizure onset (*r* = −0.49, *p* = 0.009) and social adjustment (*r* = −0.73, *p* < 0.001).

## 4. Discussion

This study’s primary aim was to assess FER in individuals with JME. FER is considered a fundamental social skill and likely plays a crucial role in social behavior in individuals with JME. Our results showed deficit tendencies in global FER and, in particular, in recognizing fear and surprise in individuals with JME. However, these results were insignificant. Nevertheless, there are apparent differences that probably would have reached the significance level with a larger sample size. In previous studies individuals with GGE showed impaired global FER and specific deficits in fear recognition [17,18,19,20]. Our results showed a similar trend and signs of additionally affected surprise recognition, which has not been previously described.

In our cohort, the individuals with JME performed significantly worse in executive function tasks than the healthy controls. These data confirm what has already emerged from previous studies, which showed impaired frontal lobe function, particularly in executive functions and attention in individuals with epilepsy [36] and JME [37,38]. While the mechanisms leading to this impairment remain incompletely understood, it is assumed that an innate thalamofrontal dysfunction with reduced connectivity between the frontal lobe and thalamus might be causal [39]. This theory is supported by the healthy siblings of individuals with JME having similar impairments [38]. However, Witt et al. showed that ASMs might also impair executive functions [40]. Interestingly we found a negative correlation between executive function and the number of ASM, supporting this theory.

This study’s secondary aim was to investigate social adjustment and personality traits in individuals with JME. Our results showed poorer social adjustment and distinct personality traits consistent with previous studies. Additionally, we showed that individuals with JME suffered more often from depressive symptoms. We found correlations between social adjustment, depression symptoms, and epilepsy variables (seizure-free interval and number of ASM), suggesting a condition caused by chronic epilepsy disease. Patients with poorly adjusted epilepsy and frequent seizures are more likely to suffer from stigmatization [41] and limitations, such as driving bans, medication side effects, and occupational restrictions, could contribute to poor social adjustment and negative emotional states. Surprisingly, we found no correlation between FER and social adjustment. Since FER is fundamental to social communication and behavior, we suspected impaired FER might be related to poor social adjustment, which was not the case in our cohort.

Interestingly, we found no correlations between FER ability and demographic variables, cognitive abilities (i.e., executive functions and IQ), depressive symptoms, or epilepsy variables. This independency indicates that a specific neuropathological process likely causes FER deficits and, unlike executive dysfunction or poor social adjustment, appears unrelated to the use of certain medications or generally to the chronic and stigmatizing condition of epilepsy. Currently, proper FER functioning is believed to depend on a reciprocal activation between the central visual processing system and an extended system of emotion processing areas, including the occipitotemporal neocortex, orbitofrontal cortex, and limbic system [21,22]. FER’s dependency on a communicating network spanning different areas rather than in a single brain region could also explain why this ability is susceptible to disturbances and is impaired in many neuropsychological diseases with different pathomechanisms, such as schizophrenia [42], Alzheimer’s disease [43], and bipolar disorder [44]. One explanation for a possible global FER impairment in individuals with JME is that this required reciprocal activation may be disrupted by altered connectivity as part of the underlying network dysfunction in JME.

Correctly recognizing emotions from facial expressions draws on a distributed structure network. While visual processing systems of the occipital and temporal lobes are responsible for emotion perception, additional knowledge is necessary for recognizing emotions [21]. Some form of memory is needed to recall learned knowledge, such as lexical labels, and to understand an emotion’s concept. For this conceptual understanding, it is crucial to feel the emotional response after viewing an emotional expression. Emotion recognition and experience are inextricably linked. The orbitofrontal cortex is a key structure in this feedback [45]. This could explain why a close reciprocal connection between the orbitofrontal and temporal cortex is needed for correct facial emotion recognition. While the orbitofrontal cortex has a key role in FER, it is just one area showing microstructural changes in individuals with JME. It shows an altered convolutional structure and volume along with changes in connectivity to various cortical and subcortical structures [8,46,47]. Therefore, it could cause impaired global FER in individuals with JME.

Another structure whose functional impairment could lead to FER deficits is the hippocampus, which is needed to recall lexical labels and provides knowledge about an emotion’s meaning. Caciagli et al. showed morphometric changes of the hippocampi and atypical mesiotemporal activation patterns during memory tasks in individuals with JME and their healthy siblings [48]. In addition, Lin et al. showed hippocampal atrophy and memory dysfunction in individuals with JME [49]. Our cohort showed signs of impaired FER, particularly for fear and surprise. Limbic structures are crucial for correctly recognizing these two emotions. The amygdala is known to be particularly involved in recognizing fear, while the parahippocampal gyrus is primarily active when recognizing surprise [50]. Changes in this area have also been found in individuals with JME. Wandschneider et al. described network isolation with reduced connectivity, mainly affecting the frontoparietal and limbic systems [12]. Similar changes were also observed in patients with bipolar disorder who showed impaired FER [51]. Therefore, altered connectivity between limbic structures and frontal cortices could be another explanation for possible FER deficits in individuals with JME.

This study’s power was limited due to its small sample size, potentially explaining the lack of significance in our findings. Further research with larger sample sizes is required to confirm a possible FER impairment in individuals with JME. Functional imaging studies could help identify causative structures. This information would be of great value in gaining further insights into JME’s pathomechanism. Further studies could also include the collection of EEG data to examine a possible relation between the degree of EEG changes and potential FER deficits. In recent years machine-learning models were build and were shown to be able to determine emotional states by analyzing EEG data [52]. By using machine-learning based analysis of EEG data, it might even be possible to objectively differentiate between typical and atypical functioning of FER and to examine a possible dependency on epileptiform discharges [53]. Bartolini et al. applied a synchronous registration of functional MRI and EEG data (fMRI-EEG) to investigate hemodynamic response to intermittent photic stimulation (IPS) in individuals with JME compared to healthy controls. They identified alterations in visual, motor, and basal ganglia circuits which were especially distinct in photosensitive individuals with JME [54]. Thus it could be from interest to distinguish between individuals with and without photosensitivity in further studies, as FER could also be affected differentially between this two subgroups of JME. Another limitation of this study was the unclear influence of ASMs on FER. Further research should include patients before or without ASM to assess its possible effects on FER. Since JME is now considered a rather heterogenic condition [55], it is also unknown to what extent interindividual differences have influenced our results. Furthermore, we could not find a correlation between FER and social adjustment. The extent to which impaired FER affects social outcomes and quality of life in individuals with JME remains uncertain and needs to be investigated in further studies.

## 5. Conclusions

This study aimed to examine FER in individuals with JME. Our results showed tendencies of impaired global FER and recognition of fear and surprise. Compared to healthy controls, individuals with JME performed significantly worse in executive function tasks and more frequently showed depressive symptoms, poorer social adjustment, and certain personality traits such as neuroticism. Several structures (i.e., the orbitofrontal cortex and limbic system) whose functionality could be impaired by the structural abnormalities previously detected in individuals with JME may potentially leading to FER deficits. Further studies are required to confirm our findings. Raising awareness about a possible FER impairment in individuals with JME could have several consequences. Difficulties in FER and social aspects should be considered as part of the neuropsychological assessment of patients with JME. Educating and raising awareness among FER-impaired individuals could help them cope with the challenges of daily life. It might even be possible to help patients improve FER with a specific course of therapy. Westerhof-Evers et al. developed a multimodal therapy protocol for patients with impaired social cognition after traumatic brain injury [56]. The protocol includes several strategies to improve FER since it is considered fundamental to social cognition. The authors showed that the therapy protocol significantly improved FER and quality of life [56]. The possibility of an effective therapy makes the knowledge of FER deficits all the more important since individuals with JME may also benefit from similar therapies to improve their social outcomes and quality of life.

## Figures and Tables

**Table 1:** Demographic and clinical characteristics.

	Individuals with JME	Healthy Controls	Statistics
N	27	27	
Sex (male/female)	12/15	12/15	*p* = 1.0 (n.s.)
Age in years (M ± SD)	25.8 ± 5.8	26.0 ± 6.1	*p* = 0.931 (n.s)
EPILEPSY CHARACTERISTICS			
Age at onset in years (M ± SD)	14.1 ± 5.1		
Seizure free interval in months (M ± SD)	26.8 ± 30.1		
Number of ASMs (M ± SD)	1.6 ± 0.8		
Most used ASMs	Levetiracetam (n = 13)		
	Valproate (n = 7)		
	Perampanel (n = 5)		
	Lamotrigine (n = 5)		
	Brivaracetam (n = 5)		

N = sample size, M = mean, SD = standard deviation, ASM = antiseizure medication, n.s. = not significant, JME = juvenile myoclonic epilepsy.

**Table 2:** Facial emotion recognition in individuals with JME and healthy controls.

	Individuals with JME	Healthy Controls	JME vs. HC	
	M ± SD	M ± SD	Z-/t-Value	*p*-Value
Ek-60FT—Global Score	44.7 ± 5.4	47.3 ± 4.3	−1.93	0.059
Ek-60FT—Happiness	9.9 ± 0.3	10 ± 0.2	−0.59	0.556
Ek-60FT—Sadness	7.1 ± 1.8	7.3 ± 1.7	−0.50	0.621
Ek-60FT—Anger	8.1 ± 1.3	8.3 ± 1.5	−0.68	0.494
Ek-60FT—Fear	5.9 ± 1.8	6.9 ± 1.9	−1.92	0.055
Ek-60FT—Disgust	5.9 ± 2.7	5.7 ± 2.1	−0.57	0.570
Ek-60FT—Surprise	7.9 ± 1.8	9 ± 1.1	−2.39	0.017

Ek-60FT = Ekman 60 Faces Task, M = mean, SD = standard deviation, HC = healthy controls, JME = juvenile myoclonic epilepsy.

**Table 3:** Neuropsychological characteristics in individuals with JME and healthy controls.

	Individuals with JME	Healthy Controls	JME vs. HC	
	M ± SD	M ± SD	Z-/t-Value	*p*-Value
*Social adjustment scale—Self Report (SAS-SR)*				
SAS-SR—global score	45.4 ± 9.5	53.9 ± 8.8	−3.38	**0.001**
SAS-SR—work	47.3 ± 12	49.9 ± 6.7	−0.35	0.729
SAS-SR—leisure and social activities	44 ± 9.8	55 ± 9	−4.28	**<0.001**
SAS-SR—relatives	45.1 ± 8.3	48.9 ± 7.8	−1.73	0.089
SAS-SR—partnership	54 ± 7.8	58.1 ± 11	−0.78	0.436
SAS-SR—parents	47 ± 6.6	53	−0.94	0.346
SAS-SR—family	49.5 ± 9.3	52.5 ± 11.7	−0.95	0.343
SAS-SR—finances	53.8 ± 8.2	57.3 ± 5	−1.64	0.102
*NEO Five-factory-Inventory (NEO-FFI)*				
NEO-FFI—neuroticism	26.3 ± 9.2	16.3 ± 8.5	4.16	**<0.001**
NEO-FFI—extraversion	25.4 ± 7.1	32.8 ± 5.5	−4.25	**<0.001**
NEO-FFI—openness to experience	27.7 ± 6.9	31.89 ± 5.87	−2.40	0.020
NEO-FFI—agreeableness	29.9 ± 5.6	34.67 ± 5.26	−3.23	**0.002**
NEO-FFI—conscientiousness	32.1 ± 9.1	35.93 ± 6.59	−1.79	0.080
CFT-20-R—IQ	98.7 ± 15.3	108.6 ± 12.5	−2.62	**0.012**
*EpiTrack*	32.6 ± 4.7	36.7 ± 3.2	−3.79	**<0.001**
*Becks depression inventory—fast screen (BDI-FS)*	4.5 ± 3.5	1.3 ± 1.8	−4.07	**<0.001**

JME = juvenile myoclonic epilepsy, HC = healthy controls, M = mean, SD = standard deviation, CFT-20-R = culture fair intelligence test, *p* value <0.05 (after correction for multiple comparisons) are in bold.

## Data Availability

The data presented in this study are available on request from the corresponding author. Data are not publicly available due to data protection reasons.
